# Assessment of genetic diversity and phylogenetic relationships in Black Pied cattle in the Novosibirsk Region using microsatellite markers

**DOI:** 10.18699/VJ21.096

**Published:** 2021-12

**Authors:** R.B. Aitnazarov, T.M. Mishakova, N.S. Yudin

**Affiliations:** Institute of Cytology and Genetics of the Siberian Branch of the Russian Academy of Sciences, Novosibirsk, Russia; Institute of Cytology and Genetics of the Siberian Branch of the Russian Academy of Sciences, Novosibirsk, Russia; Institute of Cytology and Genetics of the Siberian Branch of the Russian Academy of Sciences, Novosibirsk, Russia

**Keywords:** cattle, Black Pied breed, Novosibirsk Region, microsatellite, genetic diversity, diversity preservation, крупный рогатый скот, черно-пестрая порода, Новосибирская область, микросателлит, генетическое разнообразие, сохранение биоразнообразия

## Abstract

There are currently over a thousand indigenous cattle breeds well adapted to local habitat conditions thanks to their long history of evolution and breeding. Identification of the genetic variations controlling the adaptation of local cattle breeds for their further introduction into the genome of highly productive global breeds is a matter of great relevance. Studying individual populations of the same breed with the use of microsatellite markers makes it possible to assess their genetic diversity, relationships, and breed improvement potential. Although the Black Pied breed is the most common dairy cattle breed in Russia, there are only a few studies on genetic diversity in local Black Pied populations in some Russian regions. The goal of the present study was to analyze the genetic diversity in Black Pied cattle populations in the Novosibirsk Region and compare them with other Russian populations; to identify significantly divergent populations with a view to preserving them under the programs aimed at maintaining the genetic diversity of the domestic Black Pied breed. DNA samples from 4788 animals of the Black Pied breed from six breeding enterprises in the Novosibirsk Region have been studied using 11 microsatellite markers. No significant differences in genetic variability parameters were found between individual populations. Private alleles have been identified in five out of six populations. Five populations have shown inbreeding coefficient values (FIS) below zero, which indicates heterozygosity excess. The population distribution test, principal component analysis, FST and DEST values, cluster analysis, and phylogenetic analysis have revealed two populations genetically distinct from the others. Essentially, the genetic diversity parameters of the six studied Black Pied cattle populations from the Novosibirsk Region show no significant differences from other Russian populations of the breed. Excess heterozygosity is observed in most breeding enterprises, which is a sign of a low inbreeding rate. To maintain the genetic diversity of the Russian Black Pied cattle, we recommend focusing on the two populations with significant genetic distinctions from the others.

## Abstract (ru)

В настоящее время известно более 1000 аборигенных пород крупного рогатого скота, которые хорошо приспособлены к местным условиям среды благодаря длительной адаптации и селекции. Крайне актуально выявление генетических вариантов, контролирующих адаптацию местного скота, для переноса этих вариантов в геномы высокопродуктивных глобальных пород. Исследования отдельных популяций внутри одной породы с помощью микросателлитных маркеров позволяют оценить их генетическое разнообразие, родственные взаимоотношения и перспективы их использования для улучшения породы. Черно-пестрая порода – наиболее массовая порода крупного рогатого скота молочного направления на территории России. Однако имеются лишь единичные работы, посвященные изучению генетического разнообразия местных популяций этой породы в отдельных областях России. Целью работы являются: анализ генетического разнообразия популяций черно-пестрого скота Новосибирской области и их сравнение с другими российскими популяциями; идентификация популяций, существенно отличающихся от всех остальных, для их дальнейшего использования в программах по сохранению генетического разнообразия отечественной черно-пестрой породы. Образцы ДНК от 4788 животных черно-пестрой породы из шести племенных хозяйств Новосибирской области были исследованы по 11 микросателлитным маркерам. Значения всех показателей генетической изменчивости достоверно не различались между отдельными популяциями. Приватные аллели были обнаружены в пяти популяциях из шести. В пяти популяциях коэффициент инбридинга FIS был достоверно ниже нуля, что говорит об избытке гетерозигот. Результаты теста распределения по популяциям, анализа методом главных компонент, анализа показателей FST и DEST, а также кластерного и филогенетического анализов свидетельствуют о генетической обособленности двух популяций от всех остальных. Таким образом, параметры генетического разнообразия исследованных нами шести популяций черно-пестрого скота Новосибирской области существенно не отличаются от других российских популяций данной породы. В большинстве этих хозяйств наблюдается избыток гетерозигот, что говорит о низкой степени инбридинга. При разработке мероприятий, направленных на сохранение генетического разнообразия отечественного черно-пестрого скота, мы рекомендуем использовать животных из двух популяций, которые по генетическим характеристикам существенно отличаются от других.

## Introduction

There are currently over a thousand indigenous cattle breeds well adapted to local habitat conditions thanks to their long history of evolution and breeding (Buchanan, Lenstra, 2015). All these breeds are of high economic, scientific, historical, and cultural value (Stolpovskiy, Zakharov- Gezekhus, 2017). Meanwhile, we can see a global economically-driven replacement of local breeds by several high-productivity global breeds (Stolpovskiy, 2013). However, these breeds are typically poorly adapted to local habitats and are thus unable to reveal their outstanding qualities (Mokhov, Shabalina, 2011). As a result, identification of the genetic variations controlling the adaptation of local cattle breeds for their further introduction into the genome of highly productive global breeds is a matter of great relevance (Madan, 2005). Eventually, it will make the development of new breeds combining great productivity traits with adaptability to various geographical regions possible. For example, the H100Q mutation in gene NRAP discovered recently in Yakutian cattle seems to affect its adaptation to extreme cold (Buggiotti et al., 2021). This approach has become increasingly effective since the promising CRISPR/Cas genome editing technology was introduced into animal husbandry (Bevacqua et al., 2016; Ikeda et al., 2017).

The Black Pied breed is the most common dairy cattle in Russia (Breeds and Types of Farm Animals…, 2013). Intense breeding efforts involving the four approved Black Pied cattle types (Irmen, Priobsky, Krasnoyarsk, and Pribaikalsky) well adapted to extreme climatic conditions and local feeds are currently ongoing in Siberia (Klimenok et al., 2014). Composite cross-breeding of Black Pied cows with Holstein breeding bulls in 12 breeding enterprises in Western and Eastern Siberia has recently produced Sibiryachka, a brand new high-productivity dairy breed (Yarantseva et al., 2019).

Highly polymorphic microsatellite loci have been widely used as genetic markers in population and conservation genetics for relationship identification and other purposes (Guichoux et al., 2011; Städele, Vigilant, 2016; Galinskaya et al., 2019). In particular, microsatellites are used to analyze the origin and phylogenetic relationships of local cattle breeds (Olschewsky, Hinrichs, 2021). Studying individual populations of the same breed makes it possible to assess their genetic diversity, relationships, and breed improvement potential (Zsolnai et al., 2014; Agung et al., 2016; Szucs et al., 2019). However, studies on genetic diversity in local Black Pied breed populations are very few (Smaragdov, 2018; Modorov et al., 2021); this is especially true for the Novosibirsk Region, which remains poorly studied in this regard.

The goals of the present study were: to analyze the genetic diversity of six Black Pied cattle populations from the Novosibirsk Region and to compare them with other Russian populations; to identify significantly divergent populations to be preserved under the programs aimed at maintaining the genetic diversity of the Russian Black Pied breed.

## Materials and methods

Blood samples were taken from 4788 Black Pied cows and bulls from six breeding enterprises located in the Novosibirsk Region (referred to below as populations A–F). To analyze the populations’ structure and phylogenetic relationships, the Holstein cattle breed was used as a control group (referred to below as HOL) (van de Goor et al., 2011).

The total DNA was isolated using the COrDIS SPRINT reagent (Gordiz, Moscow, Russia) as per the manufacturer’s instructions. PCRs for 11 microsatellite markers (ETH3, INRA023, TGLA227, TGLA126, TGLA122, SPS115, ETH225, BM2113, BM1824, ETH10, BM1818) were performed using the COrDIS Cattle kit (Gordiz, Moscow, Russia) as per the manufacturer’s protocol. Fragment analysis of amplified DNA was carried out using an automated genetic analyzer NANOPHORE-05 (Syntol, Moscow, Rus-sia). The sizes of microsatellite DNA markers were calculated in GeneMapper Software 5 (Thermo Fisher Scientific, USA).

The genetic diversity parameters, F-statistics, population distribution test, and the significance of genotype distribution deviation from expected Hardy–Weinberg equilibrium (HWE) were calculated using the GenAlEx 6.5 software (Peakall, Smouse, 2012). The allelic richness (AR) was assessed via the rarefaction algorithm in HP-Rare software (Kalinowski, 2005). Calculation of the pairwise FST values and significance check of the nonzero FIS values were performed using the bootstrap method adjusted for multiple comparisons in the FSTAT software (Goudet, 2003). Cluster analysis was carried out in the STRUCTURE software (Hubisz et al., 2009). The significance of differences between populations was analyzed using Student’s t-test or one-way ANOVA with post hoc Bonferroni correction in Statistica 8.0.

The phylogenetic tree was built using the UPGMA approach based on Nei’s genetic distances in the POPTREE2 software (Takezaki et al., 2010). The statistical reliability of the phylogenetic tree was analyzed using bootstrap values based on 1000 permutations (Szucs et al., 2019). The confidence threshold was set at 70 (Lukashov, 2009).

## Results

The results of genetic variability analysis for the Black Pied cattle populations of the Novosibirsk Region can be seen in Table 1. All the microsatellite loci turned out to be highly polymorphic and contained 105 alleles in general. The average number of alleles per locus was 7.606, and the effective number – 3.604. The observed heterozygosity (0.729) was statistically similar to the expected one (0.694).

**Table 1.**
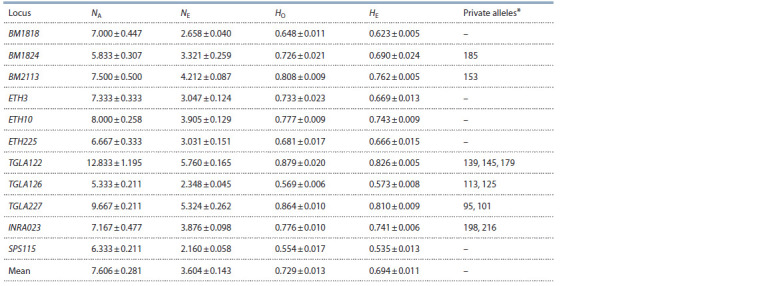
Genetic variability parameters of the microsatellite loci in the Black Pied cattle of the Novosibirsk Region (N = 4788) Notе. Here and elsewhere, the scores are given as M ± m, where M is the arithmetical mean; m is the standard error; NA is the average number of alleles per locus; NE is the number of effective alleles per locus; HO is the observed heterozygosity; HE is the expected heterozygosity; * is the unique alleles typical for a certain population.

The pairwise comparison of genetic differences for breeding enterprises, performed using Fischer’s exact test in the Genepop software, demonstrated that the cattle of each enterprise could be considered as a separate population statistically different from the others (Supplementary Material 1)1. The genetic variability parameters for each of the populations can be seen in Table 2. The maximum number of alleles per locus (8.455) was observed in population A, and the minimum one (6.273) – in population B. The allele enrichment and the effective number of alleles between populations varied between 6.087 (C) – 6.863 (F) and 3.437 (B) 3.873 (D), respectively. The observed and expected heterozygosities varied from 0.701 (F) to 0.755 (B) and from 0.682 (C) to 0.714 (D), respectively. The values of all the above indicators did not significantly differ between individual populations.

**Table 2.**
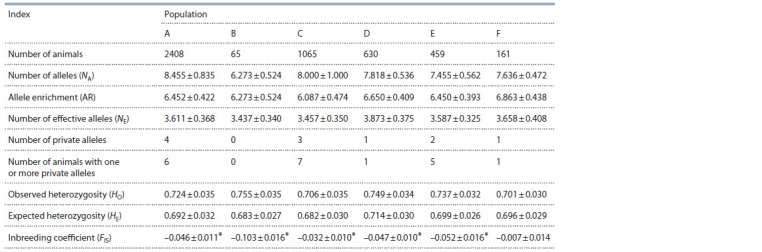
Genetic variability parameters of microsatellite loci in some populations of Black Pied cattle of the Novosibirsk Region * Fixation index ( p < 0.05) is statistically different from zero.

Supplementary Materials 1–4 are available in the online version of the paper: "http://vavilov.elpub.ru/jour/manager/files/Suppl_Aitnazarov_Engl.pdf"


The private alleles, i. e. the unique alleles characteristic for a particular animal population, were found in five of the six populations. In populations A–E, the inbreeding coefficient FIS was statistically below zero. These were the populations where in particular loci statistically significant genotype deviations from HWE were observed (Suppl. Material 2). The highest number of such loci (six) was spotted in populations A and D. Meanwhile, the genotype distribution of the ETH225 and TGLA126 loci matched HWE in all the populations investigated.

**Fig. 1.**
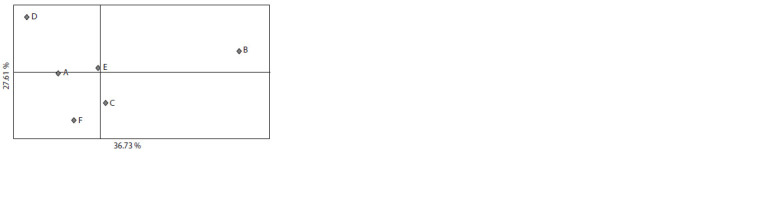
Principle component analysis results of the fixation index FST values. The axes indicate the degree of explained dispersion

Analysis of the results of a population distribution test demonstrated that, on average, 45.7 % of the animals had been properly assigned (Suppl. Material 3). However, for population B this score reached 70.8 %, which evidences this population being significantly different from the others. Principle component analysis (PCA) of the FST values showed that population B was significantly different from the others in the first component reflecting 36.73 % of the genetic variability of the whole dataset (Fig. 1). As for the second-component distribution responsible for 27.61 % of genetic variability, it was most prominent for population D.

The highest degree of genetic differentiation, both for Jost’s differentiation and the FST fixation indices, was between populations B and D (Suppl. Material 4). The closest populations in this respect were A and C.

The results of genetic clustering in STRUCTURE demonstrated that at k = 2, the population of Black Pied and HOL breeds was distributed between two different clusters (Table 3), where HOL had the highest values of similarity coefficient Q in one of the clusters. The Q values for all the Black Pied populations (except for D) were statistically lower than those for the HOL animals.

**Table 3.**
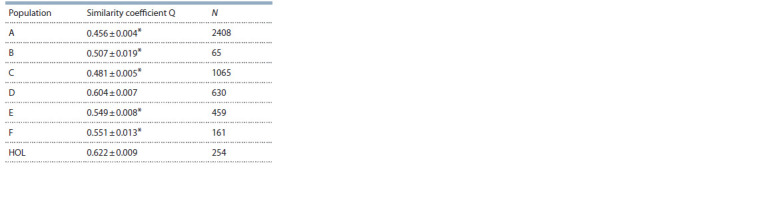
Genetic clustering results for the Black Pied (A–F) and Holstein (HOL) populations Notе. Similarity coefficient Q (Pritchard et al., 2000) was calculated for k = 2 (Q1 and Q2). Data for the HOL population were the courtesy of van de Goor et al. (2011); * is p > 0.001 for Student’s pairwise comparison against the HOL population.

**Fig. 2.**
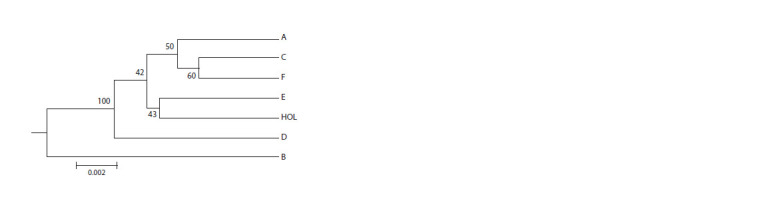
UPGMA phylogenetic tree to reveal the genetic relations between the Black Pied (A–F) (collected data) and the HOL populations (van de Goor et al., 2011). The nodes indicate the bootstrap values.

In the UPGMA phylogenetic tree built using the Nei distances, populations B and D belonged to different branches, which was statistically confirmed (Fig. 2). All the other populations including the control HOL population formed a single cluster

## Discussion

Analysis of 11 microsatellite loci from the whole sample of Black Pied cattle of the Novosibirsk Region revealed 105 alleles, which is lower than the number obtained after investigating 13 224 Holsteinized Black Pied animals in the Sverdlovsk Region (Modorov et al., 2021). The 15 loci that included the microsatellites investigated in our study contained 164 alleles, but the frequency of 38 of them did not exceed 0.1 %. On the other hand, a study of 36 animals from Poland produced just 76 alleles for a similar set of loci (10 out of 11 markers matched) (Radko et al., 2005). Thus, the observed differences may be related to the sample size and/or the number of microsatellite loci used.

The TGLA122 locus was characterized by the highest average number of alleles per locus (12.833). A similar score for this locus (14 alleles) was obtained in a study investigating the Black Pied breed from the Pskov Region (Arzhankova et al., 2015). The lowest average number of alleles per locus (5.333) was found in TGLA126, which correlates with the analogous parameter in the Black Pied breed from the Sverdlov Region (7 alleles, the frequency of 2 of them does not exceed 0.1 %) (Modorov et al., 2021). The highest (5.760) and lowest (2.160) numbers of effective alleles were detected in the TGLA122 and SPS115 loci, which also correlates with the results obtained by M.V. Modorov et al. (2021). The values of observed and expected heterozygosity (0.729 and 0.694) obtained for our sample were similar to those for the Black Pied cattle from the Sverdlovsk Region (0.73 and 0.72) (Modorov et al., 2021) but lower than the numbers for the pedigree bulls of the same breed (0.779 and 0.751) (Zinovieva et al., 2015).

It is known that the genetic data of 25–30 randomly selected animals from a population are sufficient for reliable estimation of the population’s allele frequency, expected heterozygosity and genetic distances (Hale et al., 2012). In our study, the sampling size significantly exceeded the mentioned threshold. The results of Fischer’s exact test demonstrated that all the six samples investigated could be regarded as separate populations (see Suppl. Material 1), which enabled us to shift to a more detailed analysis of their genetic differences.

Such parameters as the number of effective alleles, allele enrichment, observed/expected heterozygosity are widely used to estimate genetic variations between populations since they do not depend on a sampling size (Leberg et al., 2002; Galinskaya et al., 2019). In our study, these parameters did not have statistically significant differences between any population pairs investigated (see Table 2), which may be since all the considered breeding enterprises rely upon semen production from the same sources.

In this respect, our results are in good correlation with those of M.V. Modorov et al. (2021) who investigated 29 herds of Holsteinized Black Pied cattle from the Sverdlovsk Region and found no statistically significant genetic differences for 27 of them. Unfortunately, using microsatellite markers within a single breed for cattle population monitoring, the authors, as a rule, ignore statistical methods when comparing the genetic variability parameters (Glinskaya, 2013; Kuznetsov, 2014; Zsolnai et al., 2014; Agung et al., 2016; Szucs et al., 2019). In our study, private alleles were found in all populations, except population B, which is probably due to the size of the population (N = 65). In this respect, the Black Pied cattle from the Novosibirsk Region significantly outmatched the Black Pied animals from the Republic of Belarus, where private alleles were detected only in three populations out of nine (Glinskaya, 2013).

Inbreeding coefficient FIS is known to indicate heterozygosity reduction due to nonrandom coupling (Kuznetsov, 2014). At FIS > 0, there is a deficiency of heterozygous individuals (inbreeding); while at FIS < 0, such individuals are in excess (outbreeding). At FIS = 0, mating becomes HWE-random. In our study for most of the populations (A–E), the inbreeding coefficient was significantly below zero, meaning the heterozygotes were excessive. Consequently, populations A–E demonstrated statistically significant deviations from HWE in some of the locus genotypes (see Suppl. Material 2), which is a good correlation with the result presented above. The most probable reason for this effect might be implementation of a mating system (outbreeding; disassortative mating, etc.) aimed to reduce inbreeding (Kuznetsov, 2014). At the same time, such factors as population’s finite size, nonrandom mating, selection effect, etc. can not be completely excluded (Galinskaya, 2019).

In the population distribution test performed in our study, on average 45.7 % of animals were correctly distributed in their original groups (see Suppl. Material 3), which matched well with the 48 % distribution in a study of 16 herds of the Limousin breed in Hungary (Szucs et al., 2019). However, for population B, the distribution parameter was 70.8 %, which evidenced this population being significantly different from the others.

The results of subpopulation fixation index (FST) PCA analysis demonstrated that populations А, С, Е and F formed a compact group (see Fig. 1), for which the FST values varied from 0.004 to 0.008 (see Suppl. Material 4). Populations B and D are further from this group for the first and second components, respectively. The longest genetic distance, as per fixation index, was observed between populations B and D and comprised 0.013. The obtained genetic distances range was in good correlation with the data for single nucleotide polymorphisms (SNPs) obtained with an Illumina BovineSNP50 chip assay for the Holsteinized Black Pied cattle of six breeding enterprises in the Leningrad Region (0.002–0.012) (Smaragdov, 2018) and the populations of Jersey cattle in the USA, Canada and the UK (0.006–0.016) (Cooper et al., 2016).

According to S. Write’s classification, genetic differentiation is considered insignificant, if FST does not exceed 0.05 (Wright, 1978). However, V.M. Kuznetsov states that it is a differentiation of less than 0.01 that can be regarded as ‘insignificant and negligible’, so interpretation of the above-mentioned results can be a complex issue (Kuznetsov, 2020). Nevertheless, T.V. Galinskaya et al. assume that interpretation of the FST value is more complex than just referring to the mentioned authors (Galinskaya et al., 2019). In their opinion ‘what is more important is whether we could detect a statistically significant genetic differentiation (FST >0) or not’.

The permutation test in our study demonstrated that all the obtained FST values were statistically valid ( p < 0.01) (see Suppl. Material 4), which confirms the genetic isolation of populations B and D.

Although FST is widely used to assess genetic population differentiation, its application for multiallelic and multilocus markers such as microsatellites is often criticized (Meirmans, Hedrick, 2011; Kuznetsov, 2021). For these markers, several alternative statistical methods have been suggested such as Jost’s differentiation index (DEST), which accounts for changes in effective number of alleles (Jost et al., 2018). FST and DEST are believed to complement each other and be applied jointly (Meirmans, Hedrick, 2011; Kuznetsov, 2021). In our study, the DEST distances statistically correlated with the FST estimations (r = 0.92, p <0.0001). For both parameters, populations B and D were genetically the most distant.

Cluster analysis revealed that the Black Pied and Holstein (HOL) populations were distributed between two different clusters (see Table 3), which confirms their genetic affinity (Yurchenko et al., 2018; Yudin, Larkin, 2019). The similarity coefficient values for all the populations except D turned out to be much lower than that of HOL, which evidences different HOL pedigree levels in the investigated Black Pied populations (Zinovieva et al., 2015).

Phylogenetic analysis distributed the Black Pied populations into three groups (see Fig. 2). One group included populations А, С, Е and F that were close to HOL. Populations B and D formed two independent statistically verified branches. The result confirmed the genetic insulation of populations B and D, which we also confirmed with the results of population distribution test, PCA, FST/DEST index analysis and the results of cluster analysis presented above.

It is generally believed that to preserve a breed as a selection material, one has to sustain all its genetic pool because in most cases it is unknown which particular genes and their combination determine the economic characteristics of the breed (Stolpovskiy, 2013; Stolpovskiy, Zakharov- Gezekhus, 2017). According to the authors, the purpose of a biodiversity preservation program is to ‘sustain the diversity of the alleles a species (breed) has as well as to support the process of accumulation and potential preservation of newly appearing mutant alleles as an important source of constant evolution and improvement in animals’.

The results of the tests performed in our study confirm the genetic insulation of populations B and D from the other populations investigated, so these two populations, above all else, have to be used to preserve the genetic pool of the Black Pied breed.

## Conclusion

Thus, the genetic variability parameters of the six populations of Black Pied cattle from the Novosibirsk Region have had no significant differences from other Russian populations of this breed. Most of the breeding enterprises involved in the study have heterozygote excess due to lowlevel inbreeding. Our recommendation to those developing the programs aimed at preserving the genetic diversity of the Russian Black Pied cattle is to use animals of two populations, the genetic characteristics of which differ significantly from all others.

## Conflict of interest

The authors declare no conflict of interest.
